# An Innovative High-Strength Double-Network Hydrogel for Use as a Drilling Fluid Plugging Agent

**DOI:** 10.3390/gels10040224

**Published:** 2024-03-25

**Authors:** Yanfeng He, Jing Guo, Jinmei Bai, Le Hua, Yisong Zhang, Zhiqiang Huang, Letian Pan, Zhan Hong

**Affiliations:** School of Petroleum and Natural Gas Engineering, Changzhou University, Changzhou 213164, China; 17355251266@126.com (Y.H.); 17852423310@126.com (J.B.); s21040857017@smail.cczu.edu.cn (L.H.); s21040857001@smail.cczu.edu.cn (Y.Z.); s21040820025@smail.cczu.edu.cn (Z.H.); 2100600122@smail.cczu.edu.cn (L.P.); 13966083153@126.com (Z.H.)

**Keywords:** double-network hydrogel, water-absorbing expansion, gel strength, temperature toleration, plugging agent

## Abstract

The problem of wellbore leakage is a key challenge in the petroleum industry, limiting drilling progress and increasing drilling costs. Plugging agents play a role in repairing leaks and fractures; however, traditional plugging materials generally have low mechanical strength, poor adaptability to permeable strata, limited water absorption and expansion capabilities, and poor temperature and salt resistance. To address these limitations, a pioneering polyacrylic acid-polyacrylamide (PAA/PAM) double-network hydrogel was synthesized through aqueous solution polymerization in this study. Its strength, water absorption, expansion, temperature resistance, salt resistance, and plugging effectiveness were comprehensively evaluated. The results demonstrate that good mechanical performance is exhibited by the synthesized hydrogel, capable of withstanding a maximum stress of approximately 3.5 MPa at a 90% strain. Excellent water absorption and expansion are observed in the synthesized double-network hydrogel, with a maximum expansion of 6 times within 30 min and 8 times after 2 h. Test results show that the hydrogel had good temperature resistance and salt resistance, maintaining a strength grade E within the experimental range. The simulated evaluation of the plugging experiment indicates that, under conditions of 130 °C and 6 MPa, the leakage rate of the drilling fluid is maintained below 5 mL/min when the double-network hydrogel is utilized. From the above experimental results, it can be illustrated that excellent mechanical properties, impressive water absorption, and expansion capabilities are exhibited by the synthesized double-network hydrogel. Furthermore, the high-temperature resistance and salt resistance of the double-network hydrogel were also demonstrated. Therefore, In comparison to traditional plugging materials, significant promise is held by this newly synthesized double-network hydrogel material as a plugging agent in drilling fluids.

## 1. Introduction

Well leakage is recognized as a prevalent and intricate incident in the drilling process, with the addressing of well leaks consuming a substantial 70% of the total time dedicated to managing downhole complexities [1]. The release of harmful gases, such as methane, into the environment during well leaks elevates the risk of fire, explosions, and poisoning [2]. Additionally, it can result in groundwater contamination, soil pollution, and harm to environmental ecosystems [3]. Timely and effective well leak plugging is crucial to eliminating the release of harmful gases and halting the leakage of oil, chemicals, or other hazardous substances. Traditional plugging materials are found to have significant application defects, especially in the context of bridge plugging [4]. When faced with numerous fractures in the leaking layer, the establishment of a robust sealing layer around the wellbore by traditional plugging materials becomes challenging, potentially resulting in false sealing [5]. In the process of using cement slurry to plug leaking formations, the solidification effectiveness may be compromised due to the easy dilution of the cement slurry by formation fluids. Consequently, when confronting a significant amount of formation fluids, the plugging effect may be suboptimal or entirely ineffective [6]. Compared to traditional materials, excellent elasticity and deformation characteristics are exhibited by chemical gels, overcoming the limitations of rigid-scale grading in traditional materials [7]. They can effectively seal leakage channels of various scales [8].

Hydrogels are naturally formed or artificially synthesized polymers in an aqueous microenvironment. Within this setting, a flexible three-dimensional network structure is exhibited by these highly adaptable substances, allowing them to swell through the absorption and retention of water molecules without undergoing dissolution or structural degradation [9]. However, the mechanical properties of most natural hydrogels, such as hyaluronic acid and chitosan, are insufficient, thereby limiting their practical application [10]. To overcome this constraint, hydrogels with improved mechanical properties, such as double network hydrogels [11], interpenetrating (IPN) hydrogels [12], nanocomposite hydrogels [13], and slip-ring hydrogels [14], have been successfully developed by researchers. In 2003, the concept of double network hydrogel was first proposed by Professor Gong Jianping of Hokkaido University [15]. Due to the insufficient mechanical properties of traditional hydrogels characterized by single network structures, the introduction of a second layer in double network hydrogels has addressed this deficiency, imparting them with unique elasticity and stability [16]. Improved mechanical properties of traditional hydrogels, including strength, are significantly enhanced in the double network hydrogel, while maintaining features like biocompatibility, water absorption, and transparency [17]. Double network hydrogels are widely employed in various fields, including biological tissue engineering [18], medical materials [19], agriculture and forestry [20], environmental protection [21], sensor materials, among others. Recently, there has been an increasing level of activity in the research on double network hydrogels characterized by good biocompatibility and mechanical properties [22]. The latest advancements in the preparation of double network hydrogels encompass the creation of completely chemically cross-linked double network hydrogels [23], mixed physical/chemical cross-linked double network hydrogels [24], and fully physically cross-linked double network hydrogels [25]. Double network hydrogel is a polymer material composed of two inter-penetrating or semi-penetrating polymer networks, demonstrating superior mechanical strength or toughness compared to a single network [26]. Within double network hydrogels, the first network is typically brittle, rigid, and fully cross-linked, such as polyelectrolyte, providing sacrificial bonds during deformation, dissipating significant energy, and contributing to the mechanical strength and rigidity of double network hydrogels. In contrast, the second network is usually ductile, soft, weakly, cross-linked, or non-crosslinked, filling in the gaps of the first network and absorbing external stress, imparting flexibility to the hydrogel [27]. Therefore, these double network hydrogels offer substantial advantages in terms of mechanical properties, structure, and biocompatibility [28]. The key to the excellent mechanical properties of double-network hydrogels lies in their unique and effective network structure [29]. A physically cross-linked water-soluble gel, consisting of polyvinyl alcohol and polyacrylamide with high temperature and salt resistance, was prepared by Ma [30], showcasing substantial potential for application in reservoir stimulation. The plugging effect of the RPPG (Recrosslinkable Preformed Particle Gel) system in fractured reservoirs was evaluated by Pu [31]. At the crack, due to the delayed cross-linking between the cross-linking agent and the copolymer, cross-linking occurs at the contact interface of the gel, and the re-crosslinked RPPG exhibits better mechanical strength after swelling. Tao [32] designed a new type of water-swelling-resistant high-temperature hyperbranched crosslinkable polypropylene glycol (HT-BRPPG) for high-temperature sandstone reservoirs (130 °C) in the North Sea. This new gel system can remain stable at 130 °C for more than 15 months. Li [33] synthesized a new type of hydrogel with high thixotropy, which can be used for profile control and water plugging in horizontal wells, and can be stable for more than 90 days at reservoir temperature. Tariq [34] developed a new type of mixed double-polymer hydraulic fracturing fluid for high-temperature reservoirs, composed of guar gum derivatives and polyacrylamide-based synthetic polymers, the fracturing fluid system significantly reduced damage to the fracture surface, proppant pack, and stratum. Ying [35] prepared a polymer gel for preventing gas channeling in the killing process of high-pressure gas reservoirs, the gel can maintain high shear resistance, high temperature resistance and long-term stability at 160 °C, so it is suitable for long-term plugging of high-temperature formations. Hu [36] developed a highly elastic composite gel based on the “intercalation crosslinking” solution blending. Tao [37] designed a new polymer gel system stable at 45 °C and 65 °C for over 150 days core displacement simulation experiments and rheological tests reveal that this branched polymer has good injectivity and shear resistance in high-permeability rocks, effectively reducing the water content in the produced fluid and increasing oil production.

In this study, a novel double-network hydrogel was synthesized using polyacrylic acid and polyacrylamide as raw materials. Comprehensive testing was conducted on the synthesized hydrogel for strength, water absorption, swelling ratio, temperature resistance, salt resistance, and plugging efficiency. Through these tests, the application potential of the synthesizeddouble network hydrogel as a drilling fluid sealing agent under high-temperature and high-pressure conditions was validated.

## 2. Results and Discussion

### 2.1. Mechanical Properties of PAA/PAM Double Network Hydrogel

**Figure 1:**
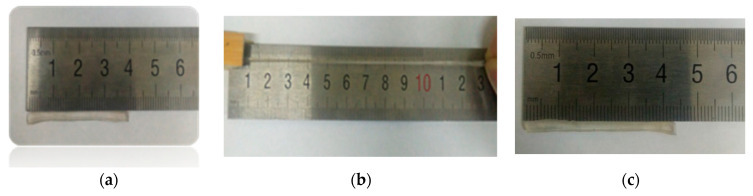
Schematic diagram of hydrogel before external force is applied (**a**), while subjected to external force (**b**), and external force is removed (**c**).

Figure 1 illustrates the length variations of the synthesized PAA/PAM double-network hydrogel under different states. The results indicate that the length of the PAA/PAM double-network hydrogel increases when subjected to external force compared to its pre-stretched state. Upon removal of the external force, the length reverts to almost the same as before stretching, demonstrating the excellent elasticity of the synthesized double-network hydrogel.

The compression test of PAA/PAM double network hydrogel was conducted using a universal testing machine, and the compression performance of PAA/PAM double network hydrogel was observed, recorded, and calculated using Formula (1). The results are shown in Table 1.

**Figure 2:**
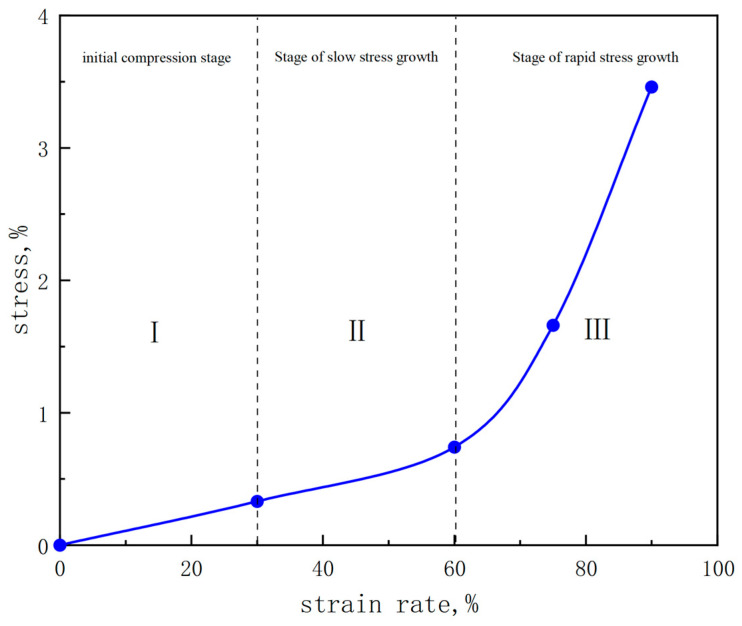
The stress-strain diagram of PAA/PAM double network hydrogels.

The strength of PAA gel is typically observed within the range of 50 to 200 KPa [38], while PAM gel strength is generally noted between 100 and 1000 KPa [39]. In contrast, the synthesized PAA/PAM double-network hydrogel demonstrates an average compressive strength of 3.57 MPa under high strain conditions, with an average strain reaching 96.3%. This exceeds the individual strengths of PAA and PAM gels, indicating a significant enhancement in strength for the synthesized PAA/PAM double network hydrogel compared to single network hydrogels. Concurrently, a series of compressive stress-strain curves were generated to observe and analyze the stress changes experienced by the hydrogel throughout the entire compression process. The results are presented in Figure 2:

It can be seen from the figure that in the initial compression stage (Stage I), with the increase of strain, the compressive stress increases relatively slowly, indicating that the gel at this stage has weak anti-compression ability. Then, as the strain continues to increase (Stage II), the magnitude strain increase is greater than that in the previous stage, indicating that the gel exhibits a higher load-bearing capacity in this stage. When the strain is close to 90% (Stage III), the stress increases greatly, which indicates that the gel can bear more stress, which is about 3.5 MPa.

### 2.2. Water Absorption and Swelling of PAA/PAM Double Network Hydrogel

As depicted in Table 2, the products underwent water absorption expansion tests, and the water absorption ratio (*Q*) was calculated according to Formula (2). It is observed that the water absorption ratio for all samples is approximately 8 times. This indicates the favorable water absorption and expansion performance of the dual-network hydrogels.

**Figure 3:**
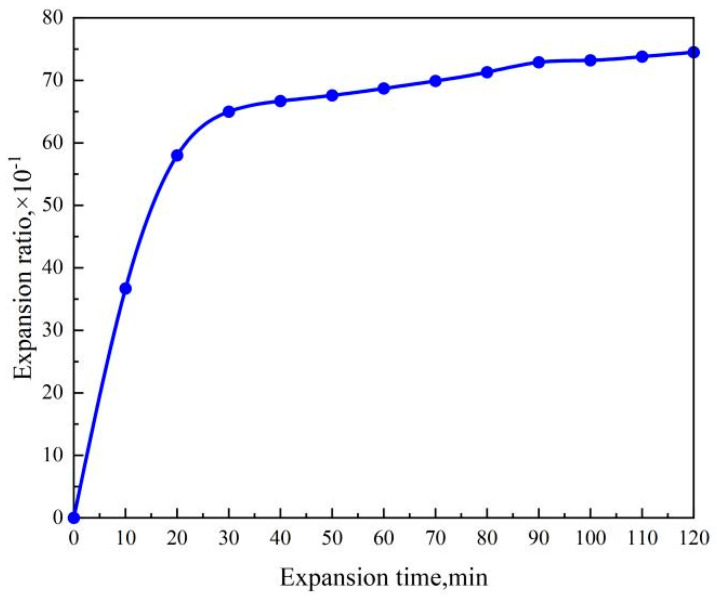
Variation diagram of water absorption expansion multiple of PAA/PAM dual network hydrogel.

Figure 3 illustrates the impact of time on the water absorption expansion ratio of the double network hydrogel. The curve in the graph illustrates a rapid expansion of the double network hydrogel before 30 min, followed by a substantial decrease in expansion rate, eventually reaching saturation. It was also noted that the water expansion ratio at 120 min (7.45) was only 1.15 times that at 30 min (6.5). When utilized as a plugging agent, the gel rapidly expands, thus significantly improving the plugging effectiveness.

### 2.3. Temperature Resistance of PAA/PAM Double Network Hydrogel

**Figure 4:**
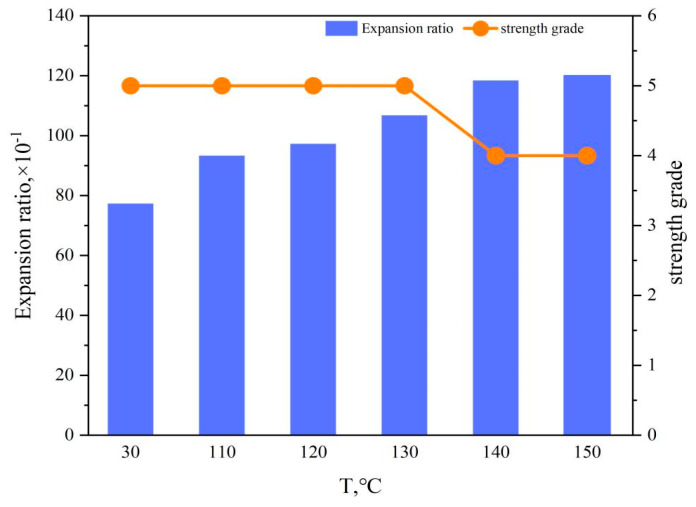
Water-absorbing expansibility and strength of PAA/PAM double-network hydrogel in distilled water at different temperatures.

Conducted high-temperature aging tests on the hydrogel, variations in water absorption rate and strength at different temperatures post-aging were examined. The test results are depicted in Figure 4.

The graph above illustrates that the gel’s expansion rate gradually increases with rising temperature, stabilizing after 140 °C, with the final expansion ratio of the double network hydrogel reaching approximately 12 times its initial volume. Prior to 130 °C, the gel strength remains at level 5 (E grade), declining thereafter.

This phenomenon is attributed to the spatial network structure of the PAA/PAM double network hydrogel being cross-linked. Before water absorption, the polymer chains of the hydrogel intertwine and cross-link with each other, forming a dense network structure that stabilizes the hydrogel overall [40]. Upon water absorption, the hydrophilic groups within the hydrogel interact with water molecules, causing the polymer chains to stretch and the network structure to expand. This expansion creates a difference in ion concentration inside and outside the network structure, generating a certain osmotic pressure that drives water molecules to permeate into the network structure [41]. After a period of water absorption, the osmotic pressure difference between the inside and outside of the polymer network structure approaches zero, and water absorption reaches equilibrium. With increasing temperature, the network structure further expands, increasing the internal space and the number of accommodated water molecules, thus absorbing more water and increasing the hydrogel’s expansion ratio [42]. Once saturation is reached, the expansion ratio of the PAA/PAM double network hydrogel remains constant. Meanwhile, the rise in temperature induces contraction and curling in the main chains and side chains of the gel molecular structure, thereby altering the properties of functional groups. This change also affects the cross-linking bonds; upon breakage of the cross-linking bonds, the cross-linking density of the PAA/PAM double network hydrogel decreases, leading to an expansion of the mesh spaces within the network structure [43]. The results in a decrease in the gel’s structural strength but facilitates the absorption of more free water, consequently enhancing the water absorption rate.

### 2.4. Salt Resistance of PAA/PAM Double Network Hydrogel

**Figure 5:**
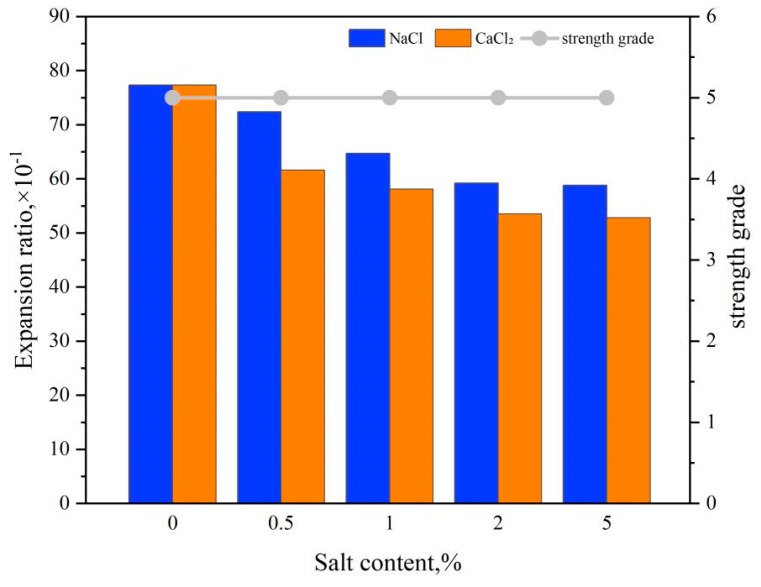
Effect of salt concentration on water absorption expansion and strength of double network hydrogels.

At 25 °C, the PAA/PAM double network hydrogel was immersed in physiological saline solutions and simulated formation waters of varying concentrations to measure the changes in water absorption rate and strength of the PAA/PAM hydrogel double network, as depicted in Figure 5.

Analyzing Figure 5 reveals a decreasing trend in the water absorption rate of the double network hydrogel with increasing salt concentration. This decrease is attributed to the presence of salt, which reduces osmotic pressure and weakens the hydration capacity of hydrophilic groups, consequently lowering the hydrogel’s water absorption capacity. Due to the greater impact of Ca^2+^ on osmotic pressure compared to Na^+^, the effect of NaCl on the hydrogel’s swelling performance is weaker than that of CaCl_2_. Experimental results demonstrate that within the range of 5–8 times expansion in water absorption for the double network hydrogel, its strength grade remains at level 5 (grade E), indicating strong resistance to NaCl and CaCl_2_ contamination.

### 2.5. Effect of Gel Plugging Agent on Viscosity of Drilling Fluid

**Figure 6:**
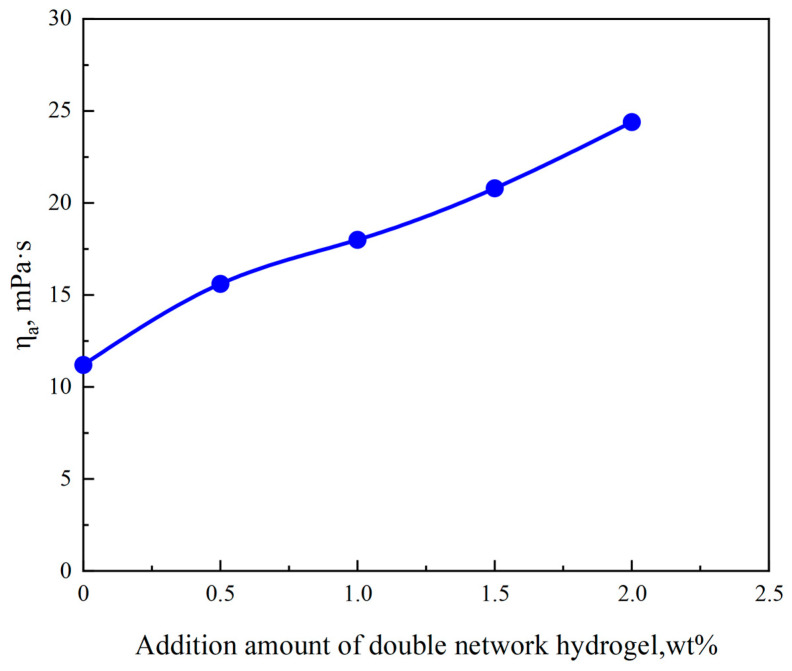
Effect of PAA/PAM double network hydrogels plugging agent on the viscosity of drilling fluid.

Figure 6 illustrates that the apparent viscosity of the drilling fluid increases with the augmentation of the double-network gel quantity. When the plugging agent’s quantity is 0.5 wt%, the viscosity of the plugging agent system escalates to 15 mPa·s. Therefore, the PAA/PAM double-network hydrogel significantly affects the rheological properties of the drilling fluid. To maintain drilling fluid stability, the plugging agent quantity should not be excessively high. An excessively high content of hydrogel can cause flocculation of clay particles in the drilling fluid, compromising the stability of the drilling fluid. Using a drilling fluid viscosity of 15 mPa·s as the evaluation criterion, the appropriate hydrogel dosage is 0.5 wt.

### 2.6. Simulation Evaluation of PAA/PAM Double Network Hydrogel Plugging

**Figure 7:**
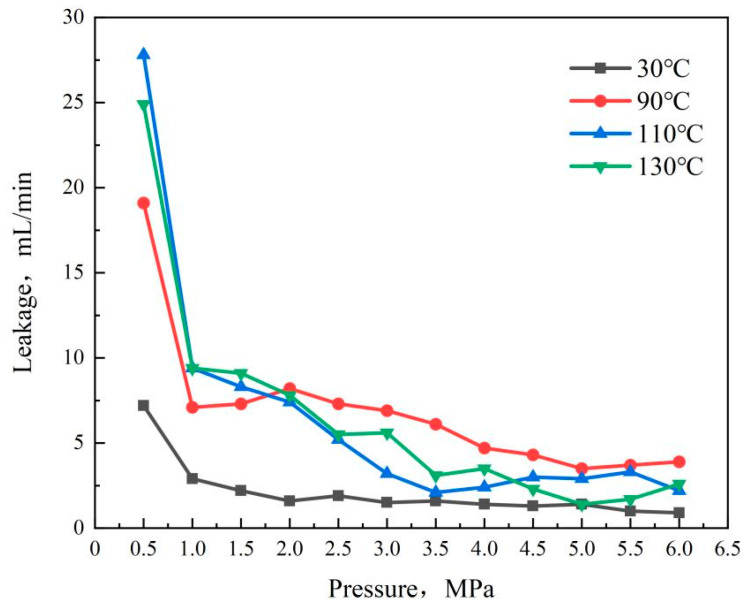
The relationship chart between the plugging effect of the hydrogel and temperature and pressure.

The change in leakage with pressure and time in Figure 7 reveals the following trends: as the hydraulic pump pressure increased during the experiment, the drilling fluid leakage was most significant when the tank pressure ranged from 0 to 1.0 MPa, resulting in the highest leakage rate. Subsequently, the drilling fluid loss rate diminished, and the loss amount gradually decreased, eventually stabilizing. This phenomenon can be attributed to the initial low system pressure, where the double-network gel particles did not enter the pores of the sand table, thus not exhibiting a plugging effect, leading to substantial drilling fluid loss. As the pressure increased, PAA/PAM double-network gel granules were pressed into the sand table pores, absorbing water and swelling, progressively reducing sand table permeability and drilling fluid leakage. At 4 MPa, the leakage rate stabilized at less than 5 mL/min, indicating a significantly enhanced plugging effect as hydrogel particles absorbed water and expanded within the sand table pores. At 6 MPa, the leakage rate remained stable below 5 mL/min, underscoring the hydrogel’s pressure-bearing plugging capability.

Comparing leakage curves at different temperatures reveals variations in the low-pressure stage at the experiment’s onset, while minimal differences emerge as the pressure reaches 4 MPa. This suggests that temperature influences the leakage rate by affecting the drilling fluid’s viscosity but does not notably impact the plugging efficacy of the hydrogel plugging agent. Furthermore, at 130 °C and 6 MPa, the plugging effect remains excellent, with a leakage rate below 5 mL/min, indicating the double-network gel plugging agent’s robust temperature and compression resistance.

## 3. Conclusions

In this study, a novel polyacrylic acid-polyacrylamide (PAA/PAM) double network hydrogel has been prepared using a solution polymerization method. The synthesized double network hydrogel has undergone tests for strength, water absorption, expansion ratio, temperature resistance, and salt resistance. Experimental results demonstrate that the synthesized double network hydrogel exhibits good strength, rapid water absorption, fast swelling, and excellent temperature and salt resistance. Furthermore, simulated plugging evaluations of the synthesized double network hydrogel revealed that under conditions of 130 °C and 6 MPa, the fluid loss of drilling fluid was below 5 mL/min. This indicates that the double network gel plugging agent possesses strong temperature resistance and compression resistance, with broad prospects as a drilling fluid plugging agent. Additionally, due to its outstanding strength, water absorption rate, and swelling ratio, the synthesized double network hydrogel can be applied in oil recovery processes for profile control and water blocking measures.

## 4. Materials and Methods

### 4.1. Materials and Instruments

Table 3 illustrates the names and sources of materials utilized in the experiment.

Table 4 illustrates the instruments utilized in the experiment.

### 4.2. Synthesis of PAA/PAM Double Network Hydrogel

**Figure 8:**
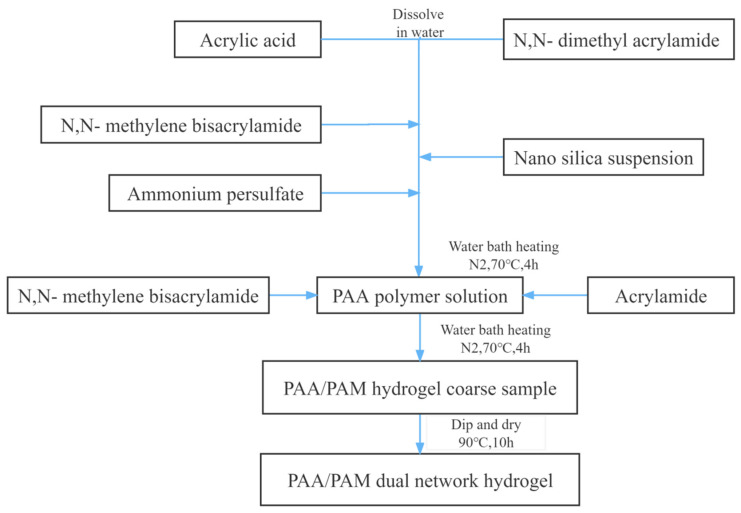
Flow chart of PAA/PAM dual network hydrogel synthesis.

As shown in Figure 8, the double-network hydrogel was synthesized through aqueous solution polymerization, employing moderate reaction temperature and facilitating control over the reaction rate. Acrylamide, acrylic acid, N,N-methylene bisacrylamide, N,N-dimethyl acrylamide, and nano-silica were utilized as raw materials, with N,N-methylene bisacrylamide serving as the crosslinking agent and ammonium persulfate as the initiator. The optimal synthesis formulation for PAA/PAM double-network hydrogel has been determined through single-factor experiments as:

(1) Monomer ratio (Acrylic acid:Acrylamide:N,N-dimethyl acrylamide) of 2:7:1;
(2) Crosslinking agent dosage of 4%:0.1%;
(3) Initiator concentration of 0.3%:0.5%;
(4) Addition of nano-silica at 40%;
(5) Monomer concentration of 20%:35%;
(6) Reaction temperature of 70 °C;
(7) pH value of 7.

**Figure 9:**
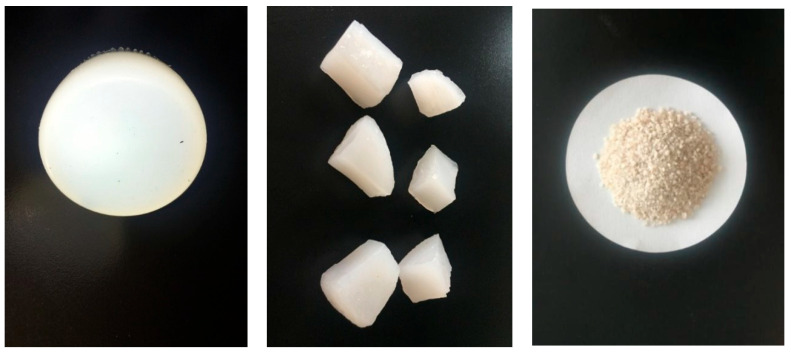
PAA/PAM double-network hydrogel and dried particles.

The appearance of the PAA/PAM double network hydrogel is depicted in Figure 9. It is evident from the figure that the synthesized double-network hydrogel exhibits a milky-white color. The surface of the synthesized hydrogel appears clear, smooth, and possesses good deformability. By altering the shape of the mold, PAA/PAM double network hydrogels with various shapes can be prepared. When subjected to manual pressure, the hydrogel maintains its integrity and is not easily broken. Additionally, its original shape is rapidly regained by the hydrogel after release, showcasing formidable elasticity.

### 4.3. Performance Test of PAA/PAM Double Network Hydrogel

#### 4.3.1. Strength of PAA/PAM Double Network Hydrogel

Utilizing the inverted bottle method, the gel strength was assessed by observing the length of the gel tongue within the bottle. The evaluation method is shown in Table 5 DSC visual code table.

When water is absorbed by the PAA/PAM double network hydrogel, its compressive performance becomes similar to that of rubber. Therefore, the compressive performance of the synthesized double network hydrogel can be demonstrated through rubber compression tests. The experimental quantification method involves cutting the hydrogel into cubes with a side length of 10 mm when the gel expansion ratio reaches 30%. Subsequently, the hydrogel is compressed using a universal mechanical testing machine at a speed of 3 mm/min, with compression being halted upon gel rupture. The compressive strength of the gel is then calculated according to Formula (1). Finally, stress-strain curves of the hydrogel are generated based on the compression test results to analyze the material’s mechanical properties [45].

(1) $$S_{s}=F/S$$

*S*: Cross-sectional area of the sample

F: Force on the cross-section

$S_{s}$: Compressive strength

#### 4.3.2. Water Absorption and Swelling of PAA/PAM Double Network Hydrogel

First, the hydrogel was prepared into dry gel with dimensions of approximately 10 mm × 10 mm × 10 mm and a mass of *m*_1_ at room temperature. Then, the dry gel was placed in a beaker, and sufficient distilled water was added. The change in swelling ratio of the hydrogel with time was recorded. Finally, after the hydrogel absorbed water and its shape stabilized, the mass of the hydrogel at this point was recorded as *m*_2_. The water swelling ratio (*Q*) was calculated using Formula (2).

(2) $$Q=\left(m_{2}-m_{1}\right)/m_{1}$$

*m*_1_: Dry gel quality

*m*_2_: Saturated hydrogel quality

*Q:* Water absorption expansion ratio

#### 4.3.3. Temperature Resistance of PAA/PAM Double Network Hydrogel

The hydrogel product was sectioned into 10 mm-sided cubes and subjected to temperature resistance testing. It underwent aging at temperatures ranging from 110–140 °C for 24 h within a vacuum drying oven. Following this, the dried products were immersed in distilled water for 48 h, surface absorption was carried out with filter paper, and subsequent weighing allowed for the comparison of gel strength. Comparisons were made between the gel strength and water absorption expansion ratio at different aging temperatures.

#### 4.3.4. Salt Resistance of PAA/PAM Double Network Hydrogel

Immersed separately in saline solutions with concentrations of 0.5%, 1%, 2%, and 5% NaCl and CaCl_2_. Salt resistance by measuring the swelling ratio and strength after water absorption.

#### 4.3.5. Effect of Gel Plugging Agent on Viscosity of Drilling Fluid

At 25 °C, a sodium-based bentonite slurry with a concentration of 4% was prepared by stirring for 2 h serving as the base slurry for drilling fluid. The prepared PAA/PAM double-network hydrogel was crushed and particles smaller than 0.9 mm were obtained through sieving with a 20-mesh (0.9 mm) screen. Different proportions of the PAA/PAM double network hydrogel were added to the drilling fluid base slurry to investigate their impact on the viscosity of the drilling fluid. The apparent viscosity of the drilling fluid was measured using a rotational viscometer.

#### 4.3.6. Simulation Evaluation of PAA/PAM Double Network Hydrogel Plugging

**Figure 10:**
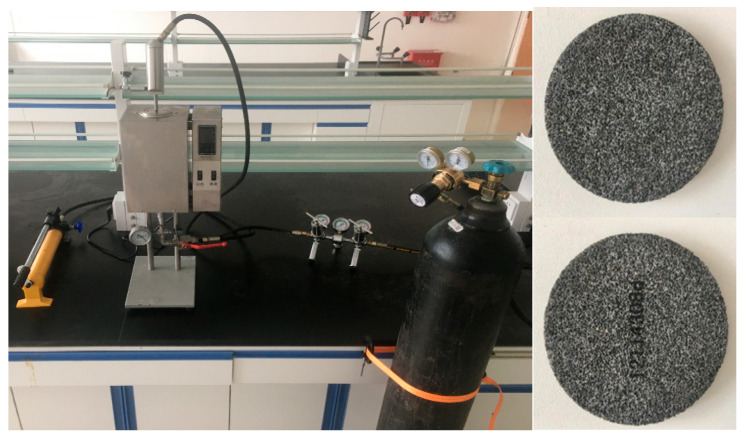
High-pressure infiltration plugging instrument and sand table.

Figure 10 depicts the high-pressure permeability plugging tester and sand table model. The simulation experiment was conducted according to the following steps:

① The drilling fluid base slurry was prepared by generating a 4% bentonite slurry, adjusting the pH to 8, and stirring for 4 h using a high-speed mixer for subsequent utilization.
② The double network gel plugging agent was formulated by crushing PAA/PAM double-network gel into particles with a size less than 0.9 mm. A specific quantity of this gel was added to the drilling fluid base slurry, stirred for 20 min with a high-speed stirrer, allowed to stand for 10 min, and then stirred for an additional 20 min for later use.
③ The sand table was selected and treated for the experiment, featuring a ceramic sand table with an average pore throat of 150 μm and a permeability of 180 D. The ceramic sand table was saturated with fresh water for 10 min prior to conducting the experiment.

## Figures and Tables

**Table 1:** Analysis table of compressive properties of dual network hydrogel.

SN	F/×10^6^ N	Ss/MPa	S/m^2^	Strain/%
1	3.63 × 10^−4^	3.63	1 × 10^−4^	98
2	3.59 × 10^−4^	3.59	1 × 10^−4^	96
3	3.68 × 10^−4^	3.68	1 × 10^−4^	97
4	3.51 × 10^−4^	3.51	1 × 10^−4^	95
5	3.46 × 10^−4^	3.46	1 × 10^−4^	99
6	3.54 × 10^−4^	3.54	1 × 10^−4^	93
Ave.	3.57 × 10^−4^	3.57	1 × 10^−4^	96.3

**Table 2:** Analysis of water absorption expansion performance of double network hydrogels.

SN	Dry Hydrogel Quality *m*_1_/g	Saturated Hydrogel Quality *m*_2_/g	Expansion Ratio of Water Absorption *Q*/×10^−1^
1	6.56	57.27	77.3
3	5.74	50.57	78.1
4	4.02	34.93	76.9
5	5.42	48.62	79.7
6	6.46	55.81	79.7
Ave.	5.75	50.16	77.3

**Table 3:** The names and sources of materials.

Material Name	Specification or Grade Abbreviation	Material Source
Acrylic acid	CP	Sinopharm Chemical ReagentCo., Ltd. (Shanghai, China)
Acrylamide	99.0%	Sinopharm Chemical ReagentCo., Ltd. (Shanghai, China)
N-N dimethylacrylamide, (DMAM)	AR	Sinopharm Chemical ReagentCo., Ltd. (Shanghai, China)
N,N-methylene bisacrylamide	CP	Sinopharm Chemical ReagentCo., Ltd. (Shanghai, China)
Ammonium persulfate	AR	Sinopharm Chemical ReagentCo., Ltd. (Shanghai, China)
Sodium hydroxide	CP	Sinopharm Chemical ReagentCo., Ltd. (Shanghai, China)
Diluted hydrochloric acid	CP	Sinopharm Chemical ReagentCo., Ltd. (Shanghai, China)
nano-silica	Particle size 30 ± 5 nm, content ≥ 99.5%	Shanghai Keyan Industrial Co., Ltd. (Shanghai, China)
N_2_	≥99.999%	--

**Table 4:** Experimental apparatus list.

Instrument Name	Specification	Instrument Manufacturer
Thermostatic water bath	HH-2	Jiayir Electric Company (Dongguan, China)
Vacuum drying oven	DZF-O	Bote Experimental Equipment Company
Electronic Balance	WT5002Y	Changzhou Wantai Weighing Instrument Co., Ltd. (Changzhou, China)
Analytical balance	--	--
Electric mixer	D90-A	Qingdao Dream Instrument Co., Ltd. (Qingdao, China)
Universal mechanical tester	CMT-6104	Shenzhen Rigol Instruments Co., Ltd. (Shenzhen, China)

**Table 5:** DSC visual code table [44].

Code	Gel State	Test Method	Gel Strength
A	Free flow type	Put the bottle upside down vertically and most of the gel flows to the bottle cap.	weak
B	Medium flow type	Put the bottle upside down vertically and 10~15% of the gel not easy flow to the bottle cap	weak
C	Difficult flow type	Put the bottle upside down vertically and the gel cannot flow to the bottle cap	medium
D	Medium deformation no-flow type	Put the bottle upside down vertically and the gel has an obvious distortion in appearance	medium
E	Small deformation static type	Put the bottle upside down vertically and the appearance of gel is slightly deformed	strong
F	Rigid type	Put the bottle upside down vertically and the gel surface is not deformed	strong

## Data Availability

The data sources in this article are all real and searchable.
